# Functional outcomes and health-related quality of life after reconstruction of segmental bone loss in femur and tibia using the induced membrane technique

**DOI:** 10.1007/s00402-022-04714-9

**Published:** 2022-12-03

**Authors:** Wolfram Grün, Emilie Johannah Jellum Hansen, Geir Stray Andreassen, John Clarke-Jenssen, Jan Erik Madsen

**Affiliations:** 1 Division of Orthopaedic Surgery, Oslo University Hospital, Kirkeveien 166, 0450 Oslo, Norway; 2 Institute of Clinical Medicine, University of Oslo, Klaus Torgårds Vei 3, 0372 Oslo, Norway; 3 Orthopaedic Department, Østfold Hospital Trust, Postboks 300, 1714 Grålum, Norway

**Keywords:** Induced membrane, Lower extremity, Masquelet, Reconstruction, Segmental bone loss

## Abstract

**Introduction:**

The induced membrane technique (IMT), frequently called Masquelet technique, is an operative, two-staged technique for treatment of segmental bone loss. Previous studies mainly focused on radiological outcome parameters and complication rates, while functional outcomes and health-related quality of life after the IMT were sparsely reported.

**Materials and methods:**

Retrospective study containing of a chart review as well as a clinical and radiological follow-up examination of all patients treated with the IMT at a single institution. The clinical outcomes were evaluated using the Lower Extremity Functional Scale (LEFS), the Short-Form-36 (SF-36) and the visual analog scale (VAS) for pain. The radiographic evaluation contained of standard anteroposterior and lateral, as well as hip-knee-ankle (HKA) radiographs.

**Results:**

Seventeen patients were included in the study. All had suffered high-energy trauma and sustained additional injuries. Ten bone defects were localized in the femur and seven in the tibia. Ten patients underwent additional operative procedures after IMT stage 2, among them three patients who contracted a postoperative deep infection. The median LEFS was 59 (15–80), and the SF-36 physical component summary (PCS) and mental component summary (MCS) were 41.3 (24.0–56.1) and 56.3 (13.5–66.2), respectively. The median length of the bone defect was 9 (3–15) cm. In 11 patients, union was obtained directly after IMT stage 2. Bone resorption was observed in two patients. At follow-up, 16 of the 17 bone defects had healed. The median follow-up was 59 months (13–177).

**Conclusion:**

Our results show a high occurrence of complications after IMT stage 2 in segmental bone defects of femur and tibia requiring additional operative procedures. However, fair functional outcomes as well as a good union rate were observed at follow-up.

## Introduction

Reconstruction of segmental bone loss represents a major clinical challenge within orthopedic traumatology. Operative techniques with vascularized fibula autograft [1] and bone transport using callotasis [2] have been used, but both techniques are associated with a long duration of treatment and frequent complications such as infections and refractures [3].

Masquelet [4] described the novel induced membrane technique (IMT) two decades ago, a two-staged surgical procedure for treatment of segmental bone loss. According to a recent systematic review [5], 48 studies reporting on 1373 patients treated with the IMT have been published. These studies have mainly focused on surgical markers of outcome, such as union and complication rates, which may not be congruent with the functional outcomes of the patients [6]. Functional outcomes after IMT in the lower extremities are sparsely reported [7–15], and to our knowledge, only one previous study [16] has reported on health-related quality of life after undergoing IMT.

The object of the current study was to evaluate the health-related quality of life and functional outcomes in patients treated for segmental bone loss in the lower extremity (femur and tibia) with the IMT at our hospital. Furthermore, we aimed to evaluate the efficacy of this surgical procedure by conducting a clinical and radiographic follow-up in addition to review the patients’ charts and imaging.

## Materials and methods

After obtaining approval from the local data protection officer, all patients treated with IMT for segmental bone loss in the lower extremity were identified by a computerized search in the hospital database.

Our inclusion criteria for the present study were segmental bone loss in femur or tibia due to acute trauma or nonunion treated with the IMT at our hospital, a minimum follow-up time of 12 months after stage 2 and age 18 years or older at the time of follow-up.

### Surgical technique

The IMT was performed as a two-staged procedure, as described by Masquelet [4]. In stage 1, thorough debridement of devitalized bone tissue was followed by implantation of an antibiotic-loaded polymethyl-methacrylate (PMMA) bone cement spacer into the bone void, and adequate soft tissue coverage and stable skeletal fixation were obtained. Within the following weeks, the cement spacer induces the formation of a vascularized, pseudosynovial membrane producing growth- and osteoinductive factors [17], and the second stage was performed approximately 6 weeks after stage one. The cement spacer was removed while preserving the membrane, the bone void filled with bone graft and the membrane closed. Autograft obtained from the iliac crest was used. If a larger volume was needed, allograft was added. Finally, wound closure was obtained [18, 19].

Postoperatively, all patients were advised partial weightbearing for 6–8 weeks. With signs of progressing radiological union present at follow-up, weightbearing as tolerated was allowed.

### Chart review

Relevant data such as age, comorbidities, smoking status, mechanism of injury, concomitant injuries, Injury Severity Score (ISS) [20], type of osteosynthesis used initially and at stage 1 and stage 2, time between stage 1 and stage 2, type of bone graft used, plastic surgical procedures for soft tissue coverage, total number of surgical procedures and the length of follow-up after stage 2 were acquired by chart review.

### Clinical examination

The clinical examination was performed by independent examinators not involved in the patients’ primary treatment. Clinical outcome scores were the Lower Extremity Functional Scale (LEFS) [21], the Short-Form-36 (SF-36) [22] and the visual analog scale (VAS) for pain. The LEFS is a reliable, valid tool for assessing functional status in patients with lower extremity musculoskeletal conditions [23] and ranges from 0 to 80 points, with 80 points representing the best possible result. The SF-36 is a quality-of-life score, consisting of eight subgroups that are used to calculate the physical and mental component summary (PCS and MCS). A higher SF-36 score reflects a better outcome. The VAS for pain both at rest and activity results in a score from 0 to 10, with 0 representing no pain and 10 representing unbearable pain. Furthermore, the 6-min walk test (6MWT) was performed, and the walking distance in meters recorded [24].

The range of motion (ROM) of the knee joint was measured in all patients using a goniometer, and ROM of the ankle joint was measured in the patients treated for tibial bone loss. To enable comparisons, measurements of the contralateral side were recorded.

Any current morbidity of the donor site from iliac crest bone graft, including affection of the lateral femoral cutaneous nerve, was documented. Clinical assessment of femoral and tibial rotation was performed with the patient in prone position and compared to the contralateral side. Any clinical axis deviation of the lower extremity was recorded.

The patients’ occupational status and return to work rate were recorded as well as the current use of walking aids, orthotics, orthopedic shoes or insoles as a consequence of the injury.

The number of surgical interventions ahead of stage one as well as the number of complications and reoperations after stage 2 was determined through chart reviews.

### Radiographic examination

Previous radiographs and CT scans were reviewed. Thereby, the original size of the bone defects was recorded, and their predominant location defined (metaphyseal or diaphyseal). In addition, current conventional anteroposterior (AP) and lateral radiographs of the femur and/or tibia as well as hip-knee-ankle (HKA) radiographs were obtained.

The conventional radiographs were used to record the presence of radiographic union at follow-up and whether union was achieved directly after IMT stage 2. Radiographic union was graded using the Radiographic Union Score for Tibial fractures (RUST) [25]. This is a score assessing the presence of bridging callus on each cortex, obtaining between 1 and 3 points per cortex. This results in a total score between 4 and 12 points, with 12 points representing bridging callus and no fracture line on any of the cortices. Union was defined as the presence of bridging callus on three of the four cortices, representing a RUST score of 10, 11 or 12 [26]. Any radiographic complication such as nonunion, bone resorption, breakage or loosening of hardware was registered, as well as any angular deformity. The HKA radiographs were used to detect and measure any lower limb length discrepancy (LLD), and any axis deviation of the lower limb toward varus or valgus was recorded. This was conducted by measuring the medial or lateral mechanical axis deviation (MAD) [27] compared to the uninjured side.

The radiographs were independently reviewed both by the first and the senior author. In cases of discrepancies, a final decision was made by consensus.

### Statistics

Parametric data are presented with means and standard deviations, while nonparametric data are presented with median and range.

## Results

Seventeen patients operated on between September 2006 and December 2020 matched our inclusion criteria and were invited to participate in a clinical and radiographic follow-up examination. All patients were willing to participate and signed an informed consent form prior to the examinations. Fifteen patients participated in a full clinical and radiographic examination at our hospital, whereas two patients, who are living abroad, answered an examination form including the clinical scores by letter and obtained current radiographs at their local hospitals.

**Fig. 1:**
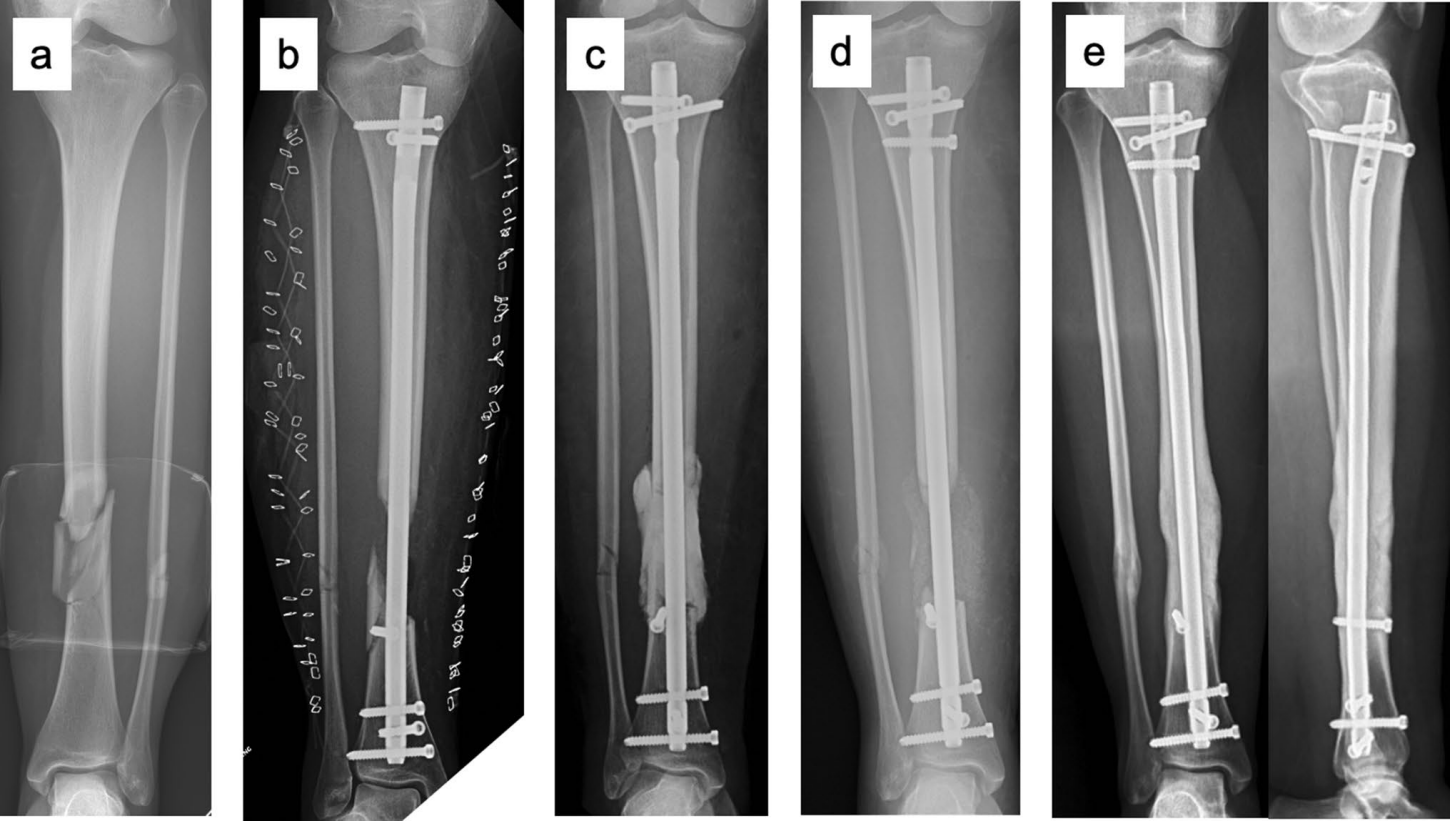
26-year-old female with a closed, segmental tibia fracture after a fall from 6 m height (**a**). After initial treatment with external fixation and fasciotomy, fragments without periosteal attachment were removed, and the fracture was stabilized with an intramedullary nail (**b**). Subsequently, the 6-cm bone void was filled with PMMA cement (**c**). After 38 days, the cement spacer was removed, and the void was filled with autologous bone graft (**d**). At follow-up 26 months after IMT stage 2, the patient had obtained good functional results and the radiographs showed complete union (**e**)

The demographic data of the study population and results from chart reviews are presented in Table 1. Sixteen patients had open fractures classified as Gustilo–Anderson III [28] and one patient initially had a closed tibial fracture but developed a compartment syndrome and was subsequently fasciotomized (Fig. 1). Eight patients had an ISS of 9 points, and the other nine patients’ ISS ranged between 10 and 29 points with a median value of 18 points. Of the 12 patients that were injured in traffic accidents, ten were involved in motorcycle accidents and two in car accidents.

**Table 1:** Demographic data of the study population and chart review^a^

Characteristic	Value
Patients	17
Sex, male/female	13/4
Age at injury^b^ (y)	44 (14–64)
Patients with diabetes	1
Smokers	6
*Etiology*	
Trauma	15
Septic nonunion	2
*Initial high-energy trauma*	17
Traffic accident	12
Fall from > 5 m height	3
Shotgun injury	2
Closed fracture	1
*Open fracture*	16
Gustilo–Anderson IIIA	7
Gustilo–Anderson IIIB	7
Gustilo–Anderson IIIC	2
*Localization*	
Femur	10
Tibia	7
Metaphyseal	8
Diaphyseal	9
Patients with concomitant injuries	17
Injury Severity Score (ISS)^b^	10 (9–29)
*Definite fixation type*	
Intramedullary nail	10
Plate	7
Time between injury and IMT stage 1^b,c^ (d)	13 (0–39)
Time between IMT stage 1 and 2^b^ (d)	44 (21–93)
Surgical procedures prior to IMT stage 2^b,d^	5 (1–12)
*Bone graft type*	
Iliac crest autograft	17
Adjuvant allograft	9
Plastic surgery for soft tissue closure	8
Free muscle flap	6
Additional surgery after IMT stage 2	10
Additional surgery for major complication	8
Additional surgery to obtain union	6
Surgical procedures until union^b,d^	6 (3–20)
Follow-up^b^ (mo)	59 (13–177)

^a^Numbers are presented as number unless otherwise indicated

^b^Numbers are presented as median (range)

^c^Patients with traumatic bone loss

^d^Affected part of the extremity

**Fig. 2:**
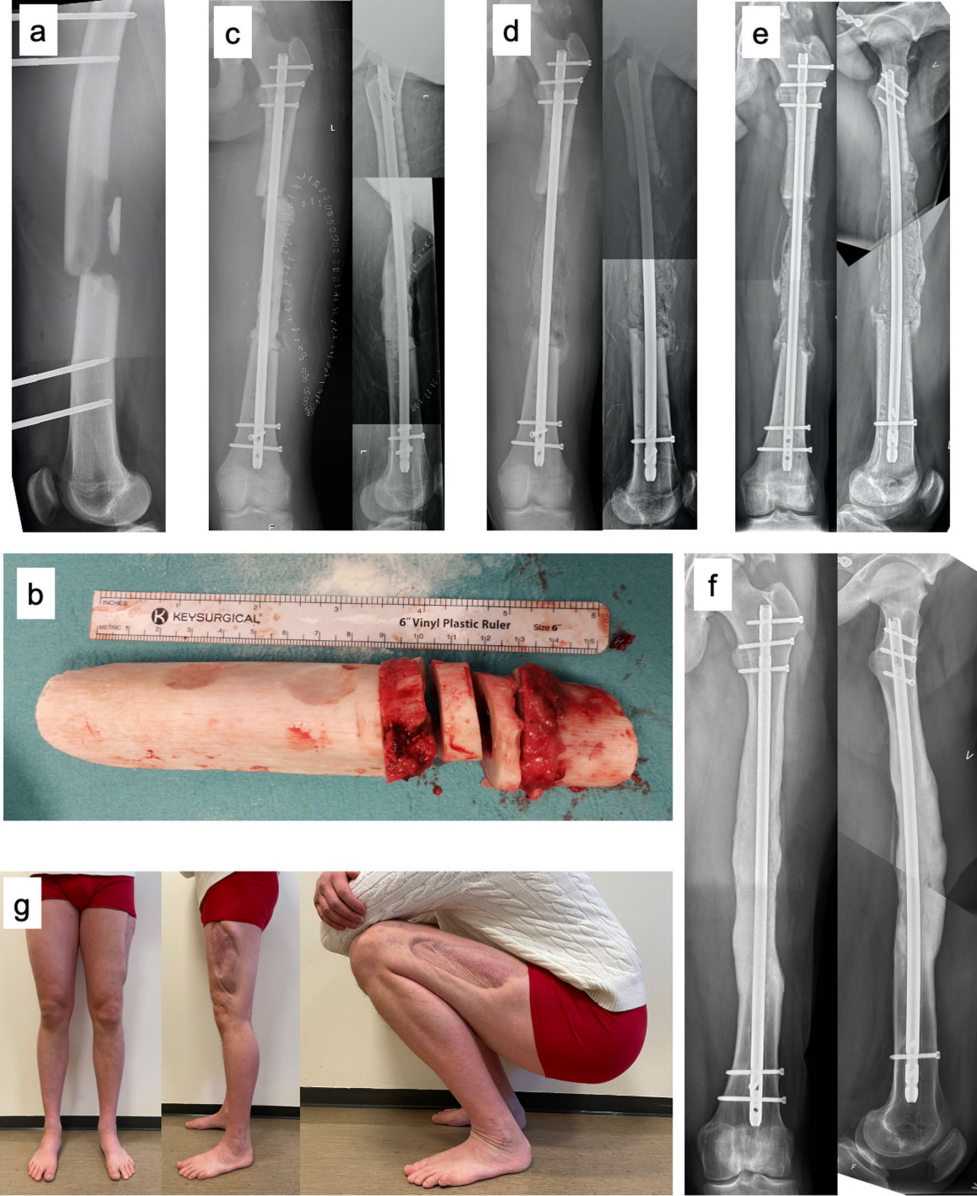
17-year-old male who sustained a Gustilo–Anderson IIIB open femoral fracture and an open book pelvic injury in a motorcycle accident. After initial external fixation (**a**), the femoral fracture was stabilized with a retrograde intramedullary nail. However, the patient developed a fulminant infection leading to removal of the nail and repeated external fixation. He had positive wound cultures for E. coli, Bacteroides, Clostridia and Staphylococcus capitis and was treated with antibiotics (Penicillin, Ciproxin, Metronidazole and Linezolid) in addition to repeated wound irrigation and debridement. Eventually, 15 cm of devitalized femur had to be removed (**b**). After 11 surgical procedures on the femur and 30 days after the initial injury, the femur was stabilized with an antegrade intramedullary nail, and the bone void was filled with a gentamicin loaded PMAA cement spacer (**c**). 31 days later, IMT stage 2 was performed, and the bone void was filled with bilateral iliac crest autograft, blended with adjuvant allograft (**d**). Radiographs taken 6 weeks postoperatively show incipient ossification of the graft (**e**). At follow-up 24 months later, the patient presented with a good functional result and the radiographs showed good ossification of the graft (**f**, **g**)

Of the 15 cases with traumatic bone loss, 13 were initially stabilized with external fixation and received their final stabilization type at IMT stage 1 (Fig. 2), whereas two patients initially were operated on with plate and intramedullary nail, respectively, without undergoing later implant changes. In the two patients with septic nonunion, the fixation type was changed from plate to intramedullary nail at IMT stage 1. Fifteen patients underwent more than one surgical procedure (median 6, range, 2–12) prior to IMT stage 2.

An overview of the major complications is given in Table 2. A total of eight patients were reoperated due to major complications that occurred after IMT stage 2. Of these, six patients had bone defects ranging from 9 to 15 cm, while only two had defects of eight centimeters or below. Three patients developed a deep surgical site infection after IMT stage 2; one patient with femoral bone loss was treated with removal of the bone graft, repeated wound irrigation, and implantation of a new cement spacer after infection control was obtained. Bridging callus developed rapidly around the spacer leading to the decision not to perform a new bone grafting procedure, and the spacer was left in place. The second patient was treated with removal of a tibial nail and subsequent stabilization with a Taylor Spatial Frame (TSF) until infection control and union were obtained. The third patient was treated with repeated wound irrigation and antibiotics, and the infection resolved. No limbs were amputated.

**Table 2:** Major complications (*n*)

*Deep infection (positive wound cultures; requiring revision surgery)*	8
Prior to IMT stage 2	3
After IMT stage 2	3
Septic nonunion prior to IMT	2
Knee joint stiffness	5
Malrotation	3
Reoperation for malrotation	1
Resorption of graft	2
Hardware breakage	2
Refracture	1
Pulmonary embolism	1
Ilioinguinal nerve neuropraxia and os ilium fracture after bone harvesting	1

Two patients with knee joint stiffness have later been operatively treated with a Judet quadricepsplasty [29]. One of these patients has also been operatively treated with a proximal femoral osteotomy due to a femoral internal malrotation of 30 degrees and had a normal clinical rotation at follow-up. In two other patients, we recorded a clinical external malrotation of 10 and 30 degrees, respectively. However, they did not require operative correction of the malrotation.

**Fig. 3:**
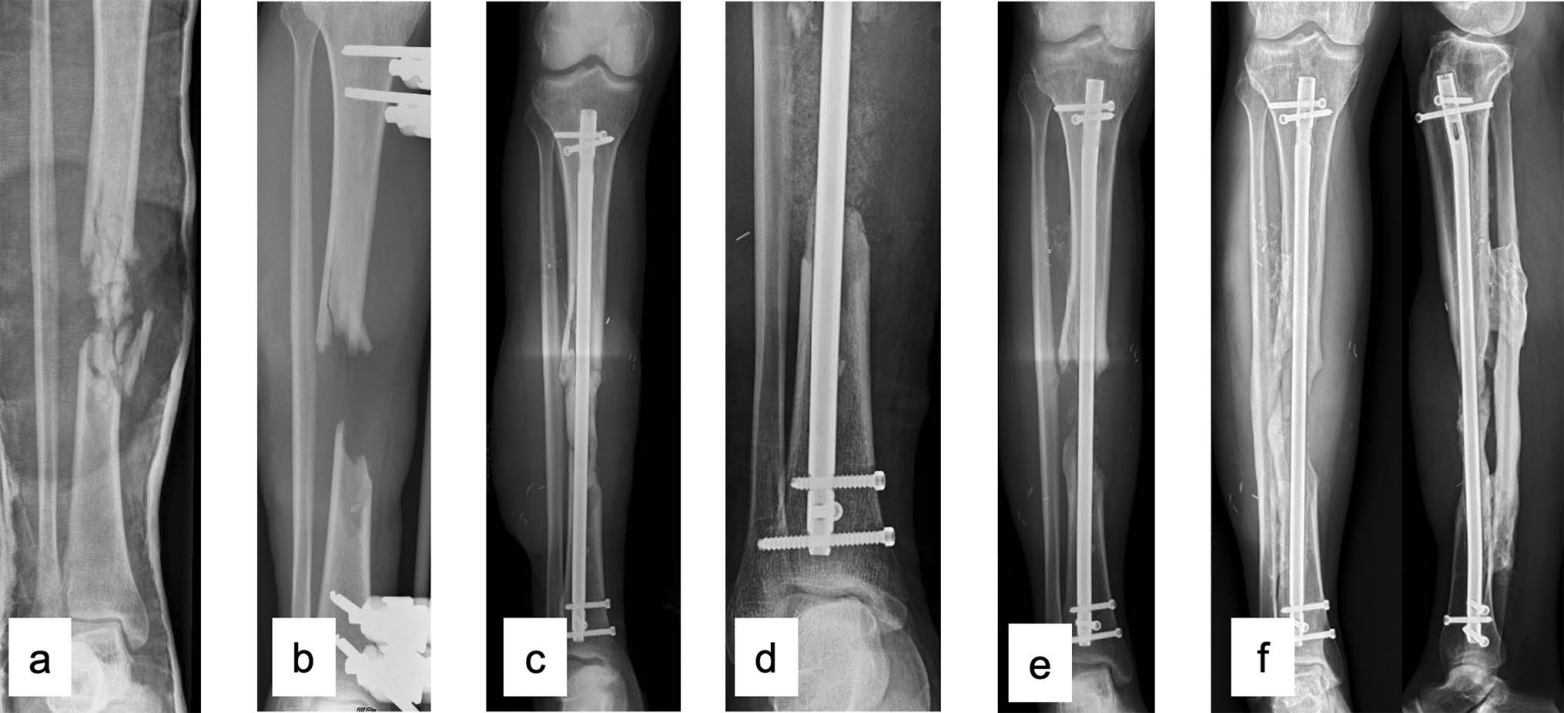
47-year-old male with a Gustilo–Anderson III B shotgun injury, presenting at our department 10 days after injury (**a**). Debridement, removal of loose bone fragments, and external fixation were performed (**b**). The patient had positive wound cultures for Enterococcus cloacae and was treated with antibiotics in addition to repeated revision surgery. Twenty-four days after injury, intramedullary nailing of the fracture was performed, and the 9-cm bone defect was filled with a cement spacer in addition to soft tissue closure with a free muscle flap and skin grafting (**c**). IMT stage 2 was performed 37 days later (**d**). However, radiographs taken one year after injury showed resorption of the graft (**e**). Subsequently, a decorticating procedure of the tibia and fibula in addition to autologous bone grafting and implantation of bone morphogenetic protein (BMP2) was performed. Radiographs obtained at follow-up 69 months after the initial treatment show a tibiofibular bone-bridge both proximal and distal to the bone defect (**f**)

In two patients, resorption of the bone graft occurred. One of them was reoperated with a one-stage decorticating procedure of the tibia and fibula in addition to autologous bone grafting and implantation of bone morphogenetic protein (BMP2). Thereby, a tibiofibular bone-bridge both proximal and distal of the bone defect was obtained (Fig. 3). The other patient, a smoker, sustained a new trauma 9 weeks after stage 2 which resulted in a bent femoral plate and was subsequently reoperated with implantation of a new plate. Nonunion and loosening of the plate were observed 7 months after stage two, and the patient was reoperated with revision of the nonunion, femoral shortening and plate osteosynthesis, and subsequently obtained union.

One patient underwent femoral plate removal 37 months after IMT stage 2, having obtained union. Even so, 16 months later he sustained a refracture at the site of the initial bone defect which subsequently was treated with an intramedullary nail and healed uneventfully.

The results of the clinical follow-up examinations are presented in Table 3. One 69-year-old patient followed up 59 months after IMT for a septic femoral nonunion reported on a significantly decreased general health status due to reasons unrelated to the orthopedic subject. This may have affected this patient’s poor clinical outcome (LEFS 15, 6MWT 25 m, SF-36 PCS 24.0 and SF-36 MCS 25.6) despite having obtained union and a RUST score of 12. Seven patients reported on donor site morbidity from the iliac crest; four patients had impaired skin sensation including one patient with a lateral cutaneous femoral nerve injury; and three patients reported on pain from the donor site.

**Table 3:** Clinical outcome measures^a^

Characteristic	Value
LEFS	59 (15–80)
*SF-36*	
Physical function (PF)	75 (5–90)
Role physical (RP)	50 (0–100)
Bodily pain (BP)	57.5 (0–100)
General health (GH)	70 (5–95)
Vitality (VT)	70 (5–90)
Social function (SF)	62.5 (12.5–100)
Role emotional (RE)	100 (0–100)
Mental health (MH)	84 (16–96)
Physical component summary (PCS)	41.3 (24.0–56.1)
Mental component summary (MCS)	56.5 (13.5–66.2)
VAS at rest	1 (0–6)
VAS at activity	3 (0–8)
6-min walk test (6MWT) (m)	480 (25–780)
*Range of motion (ROM)*	
Injured knee ROM (degrees)	125 (80–170)
Uninjured knee ROM (degrees)	140 (125–160)
Injured ankle ROM (degrees)	35 (28–65)
Uninjured ankle ROM (degrees)	52.5 (42–66)
Iliac crest donor site morbidity^b^	7
*Occupational status*^b^	
No change in occupation	4
No change in occupation, but partially disabled	3
Change in occupation	4
Disabled	6
Use of orthopedic aids^b^	5

^a^Numbers are presented as median (range) unless otherwise indicated

^b^Numbers are presented as number

The radiological results are presented in Table 4. The only patient not having achieved union is illustrated in Fig. 3; however, a functional fibula pro tibia union had been obtained. Union was obtained in 11 patients without additional surgical procedures after IMT stage 2.

**Table 4:** Radiological outcome measures^a^

Characteristic	Value
Length of bone defect (cm)	9 (3–15)
Union directly after IMT stage 2^b^	11
Union at follow-up^b^	16
RUST at follow-up	12 (8–12)
MAD discrepancy compared to uninjured side^b^	12
MAD toward valgus^b^	6
MAD difference (mm)	14 (5–26)
MAD toward varus^b^	6
MAD difference (mm)	7.5 (3–43)
Limb length discrepancy (mm)	– 6.5 ( – 108–3)

^a^Numbers are presented as median (range) unless otherwise indicated

^b^Numbers are presented as number

In 14 patients, the HKA radiographs revealed a limb length deficiency, six patients had a deficiency of 10 mm or more (median 24 mm, range, 15–108). Only three of these patients were using a shoe lift at follow-up.

Three of the seven tibial defects healed with a slight procurvatum (range, 2–7 degrees). Coronal malalignment was found in five patients. One femur showed a valgus malalignment of 3 degrees, whereas 3 femurs had a varus malalignment of 7, 13 and 13 degrees, respectively. One tibia showed a varus malalignment of 2 degrees.

## Discussion

Our results demonstrate that the IMT is a limb-saving operative procedure for treating potentially limb-threatening injuries. All 17 patients from the current study initially sustained high-energy injuries with the vast majority being open fractures. At follow-up, all but one patient had obtained union and the functional results were fair.

We have performed the IMT after Masquelet’s recommendations [4], with a median interval between IMT stage 1 and 2 of 44 days, which is in accordance with the 6–8 weeks recommended by most authors [18, 30, 31]. In select cases where incipient bony callus surrounding the cement spacer was observed at an earlier time point, IMT stage 2 was performed prior to the 6 weeks mark. This was observed particularly in young patients (Fig. 2).

In the current study, 11 of the 17 patients (65%) obtained union directly after IMT stage 2, without further operative interventions. This is somewhat less than previously reported; in their systematic review including 48 studies and 1386 cases, Fung et al. [5] found a union rate of 82% after IMT stage 2, without additional procedures, and Mi et al. [32] reported on a union rate of 89% after IMT stage 2. One reason for this difference might be that the bone defects in our series (median 9 cm) were larger than in these reviews (5.9 cm and 6.3 cm, respectively).

At follow-up, 16 of our 17 patients (94%) had radiographically confirmed union according to the RUST scale. This compares well with Fung et al. [5] and Mi et al. [32] reporting union rates of 88%, and 92%. Our single patient not having obtained union of the defect had obtained bony stability via tibiofibular bone bridges (Fig. 3).

The follow-up time in the present work varied from one year to almost 15 years with a median of 5 years, longer than in Morelli et al.´s review of 17 papers, with a mean follow-up time of 16 months [33]. Even if shorter follow-up times might be sufficient to report on the rates of union and major complications following the IMT, a longer follow-up time enables evaluation of long-term consequences of the injury and treatment, like the return-to-work rate, health-related quality of life after completed rehabilitation, and the eventual onset of late sequelae like posttraumatic osteoarthritis.

Of the major complications, we found deep infection to be the most frequent. Three of the 15 patients with traumatic bone loss and the two patients with previously infected nonunions suffered a deep infection prior to IMT stage 1 (33%). None of these patients, however, experienced recurrence of the infection at a later stage. Our rate of infections is lower than previously described; Fung et al. [5] reported a 60% infection rate prior to the IMT in a systematic review of 48 papers including 1386 patients [4]. Their high numbers might be caused by a higher share of infected nonunions and post-traumatic osteomyelitis. After IMT stage 2, three other patients developed deep infections (18%) in our study, compared with an infection rate of 21% in Giotikas et al.’s study [34] of 14 fractures with traumatic bone loss after mainly Gustilo–Anderson grade III open fractures [35]

Most previous studies on the IMT focus on the radiological outcomes as well as the incidence of complications. These parameters may not reflect the patients’ subjective experience of their outcome. Only one previous study has evaluated the health-related quality of life after IMT, using the SF-12 [16], which is a reduced size version of the SF-36 but provides comparable results regarding the PCS and MCS [35]. The authors investigated the outcomes of 150 atrophic and/or infected nonunions treated with the IMT and reported a PCS of 36.7 (16.9–56.6) and a MCS of 48.7 (22.3–68.3) 12 months postoperatively. The authors evaluated lower scores compared to our study (PCS 41.3 and MCS 56.5), maybe due to their relatively short follow-up time and reported consolidation rate of only 80% at last follow-up.

A few other studies have reported on clinical outcome scores and PROMs (Patient-reported outcome measures) after the IMT, including three studies reporting mean LEFS values from 53–68 after 23–32 months follow-up [12–14]. In our study, the LEFS was 59 and ranged from a poor 15 points to the optimum of 80 points. Our results seem to be comparable with these previous reports; however, none of the mentioned studies report about their patients’ additional injuries. We consider this a necessity since additional injuries might affect the healing potential and prognosis of the IMT, the patients’ general rehabilitation potential and thereby their general clinical and subjective outcomes. To illustrate this, two of our patients with tibial traumatic bone loss had unsatisfactory clinical outcomes with LEFS scores of 23 and 26. One had an ipsilateral open femoral fracture, and the other patient had a talus fracture and metatarsal fractures compromising their lower extremity function, demonstrating the shortcomings of the LEFS in reflecting the isolated functional outcomes after a bone defect reconstruction.

Recently, Biz et al. [36] reported on the functional outcome and complications 15 to 30 years after treatment of comminuted tibial fractures or deformities using Ilizarov bone transport to cover bone defects of mean 7 cm. In the open fracture patients, the authors reported a mean LEFS of 19, which is lower than in our study. In comparison, their patients treated for deformity had a mean LEFS of 77. However, the authors reported the range of the LEFS from 0 to 100, which originally ranges from 0 to 80 [23, 37]. 25% of their patients suffered complications requiring additional surgery, which is a lower rate than in our study, but their routine use of bone grafting of the docking site was not counted herein.

Numerous previous studies report on the time to union after the IMT. For the current study, we chose not to include that parameter due to the study’s retrospective design; as the frequency and intervals of radiographic follow-up examinations have varied among our patients, we were not able to provide valid data regarding time to union.

Despite the circumstance that all our patients had potentially limb-threatening injuries with partly multiple additional injuries and the frequent occurrence of complications and additional surgery, none required a limb amputation. Amputation is a regularly reported endpoint after segmental bone loss in the lower extremity. Morris et al. [38] report on amputations in two of their 12 patients treated for tibial bone loss, Morelli et al. [33] evaluated an amputation rate of 4% in their review, Mi et al. [32] found amputation in 3% of their patients, while Biz et al. [36] reported a 5.5% amputation rate after Ilizarov bone transport.

Our study has some inherent weaknesses and shortcomings. It is retrospective, does not have a control group and consists of a relatively low total number of patients. Therefore, a statistical analysis consisting of a regression analysis was not feasible. Furthermore, the indication for IMT was heterogenous, the follow-up time varied from one to 15 years, and most of our patients have suffered multiple injuries leading to sequelae possibly influencing our main outcome measures. Thus, our clinical and functional results might have been influenced by other factors than the IMT procedure and must be interpreted in that light.

The strengths of our study are that we have obtained both a radiological and clinical follow-up including functional and quality-of-life scores of all patients that have been treated with IMT at our institution. A homogenous operative technique has been applied, and all patients have been clinically assessed by the same independent examinators.

## Conclusion

The IMT is a limb-saving operative technique for the treatment of segmental bone loss. We found the procedure to be associated with a high rate of complications and additional operative procedures. However, no amputations were required, and the reported clinical outcomes as well as health-related quality of life can be considered acceptable in light of the primary injuries’ severity and entailing challenges.
